# X-Linked Hypophosphatemic Rickets: Multisystemic Disorder in Children Requiring Multidisciplinary Management

**DOI:** 10.3389/fendo.2021.688309

**Published:** 2021-08-06

**Authors:** Giampiero Igli Baroncelli, Stefano Mora

**Affiliations:** ^1^Pediatric and Adolescent Endocrinology, Department of Obstetrics, Gynecology and Pediatrics, University Hospital, Pisa, Italy; ^2^Laboratory of Pediatric Endocrinology and Bone Densitometry Service, IRCCS San Raffaele Scientific Institute, Milan, Italy

**Keywords:** burosumab, conventional treatment, management, X-linked hypophosphatemic rickets, complication

## Abstract

X-linked hypophosphatemic rickets (XLH) is the commonest inherited form of rickets. It is caused by an impaired regulation of fibroblast growth factor 23 (FGF23) due to a PHEX gene mutation, which leads to reduced tubular reabsorption of phosphate and renal 1α-hydroxylase activity and increased renal 24-hydroxylase activity. Hypophosphatemia associated with renal phosphate wasting, normal serum levels of calcium, parathyroid hormone, and 25-hydroxyvitamin D represents the main biochemical sign in affected patients. Patients with XLH show rickets and osteomalacia, severe deformities of the lower limbs, bone and muscular pain, stunted growth, and reduced quality of life. However, XLH is a multisystemic disorder requiring multidisciplinary approaches in specialized subdisciplines. Severe complications may occur in patients with XLH including craniosynostosis, hearing loss, progressive bone deformities, dental and periodontal recurrent lesions, and psychosocial distress. Moreover, long-term conventional treatment with active vitamin D metabolites and oral inorganic phosphate salts may cause endocrinological complications such as secondary or tertiary hyperparathyroidism, and adverse events in kidney as hypercalciuria, nephrocalcinosis, and nephrolithiasis. However, conventional treatment does not improve phosphate metabolism and it shows poor and slow effects in improving rickets lesions and linear growth. Recently, some trials of treatment with recombinant human IgG1 monoclonal antibody that targets FGF23 (burosumab) showed significant improvement of serum phosphate concentration and renal tubular reabsorption of phosphate that were associated with a rapid healing of radiologic signs of rickets, reduced muscular and osteoarticular pain, and improved physical function, being more effective for the treatment of patients with XLH in comparison with conventional therapy. Therefore, a global management of patients with XLH is strongly recommended and patients should be seen regularly by a multidisciplinary team of experts.

## Introduction

X-linked hypophosphatemic rickets (XLH, MIM 307800) is the most common inherited form of rickets (1) with a prevalence of 1:20,000-60,000 (2, 3). XLH is caused by alterations in the gene coding for phosphate regulating endopeptidase homolog X-linked (PHEX), a protein regulating the expression of fibroblast growth factor 23 (FGF23). High serum FGF23 concentration impairs renal reabsorption of phosphate and 1α-hydroxylase activity, and stimulates renal 24-hydroxylase activity. This leads to hypophosphatemia and low or inappropriately normal serum 1,25-dihydroxyvitamin D [1,25(OH)2D] concentration in the setting of hypophosphatemia (4, 5). Several other genes, such as *FGF23*, *DMP1*, and *ENPP1*, are involved in the synthesis, signaling, and regulation of FGF23 and their mutations are responsible for hypophosphatemia; they account for less than 20% of the cases (6).

## Clinical Phenotype

**Figure 1:**
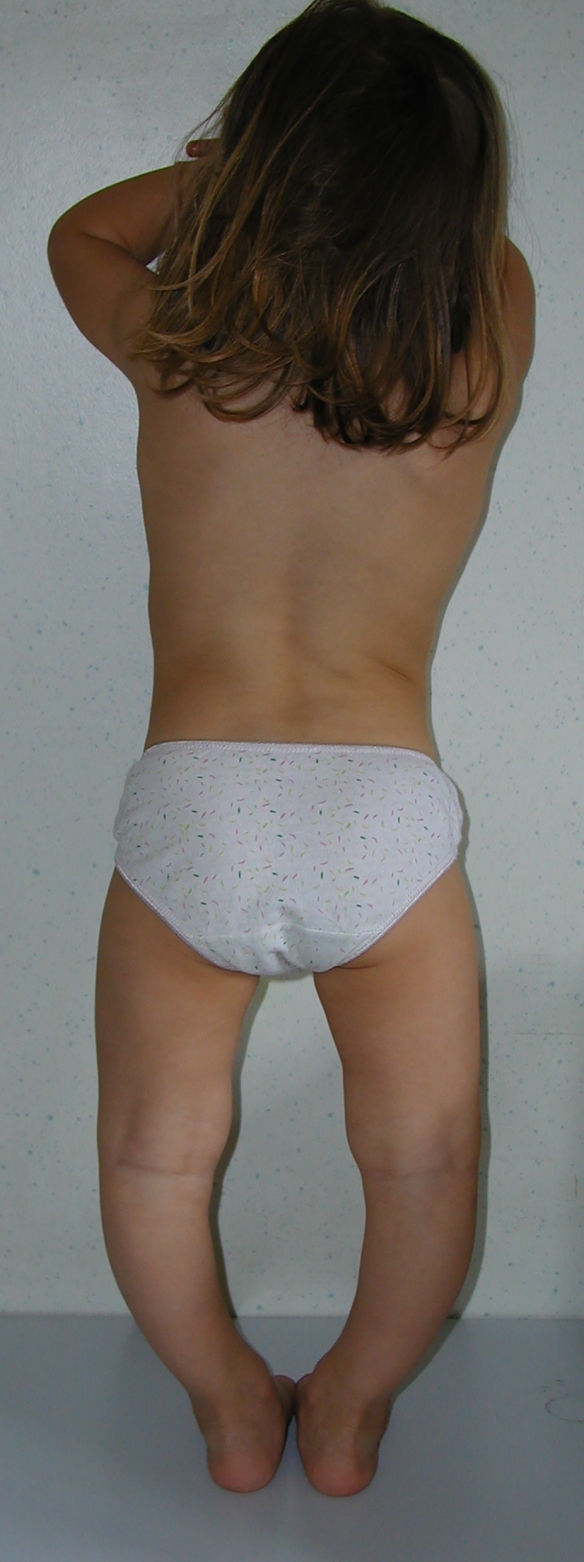
Severe genu-varum in a 3.2-year-old female patient with XLH at diagnosis with disproportionate short stature.

**Figure 2:**
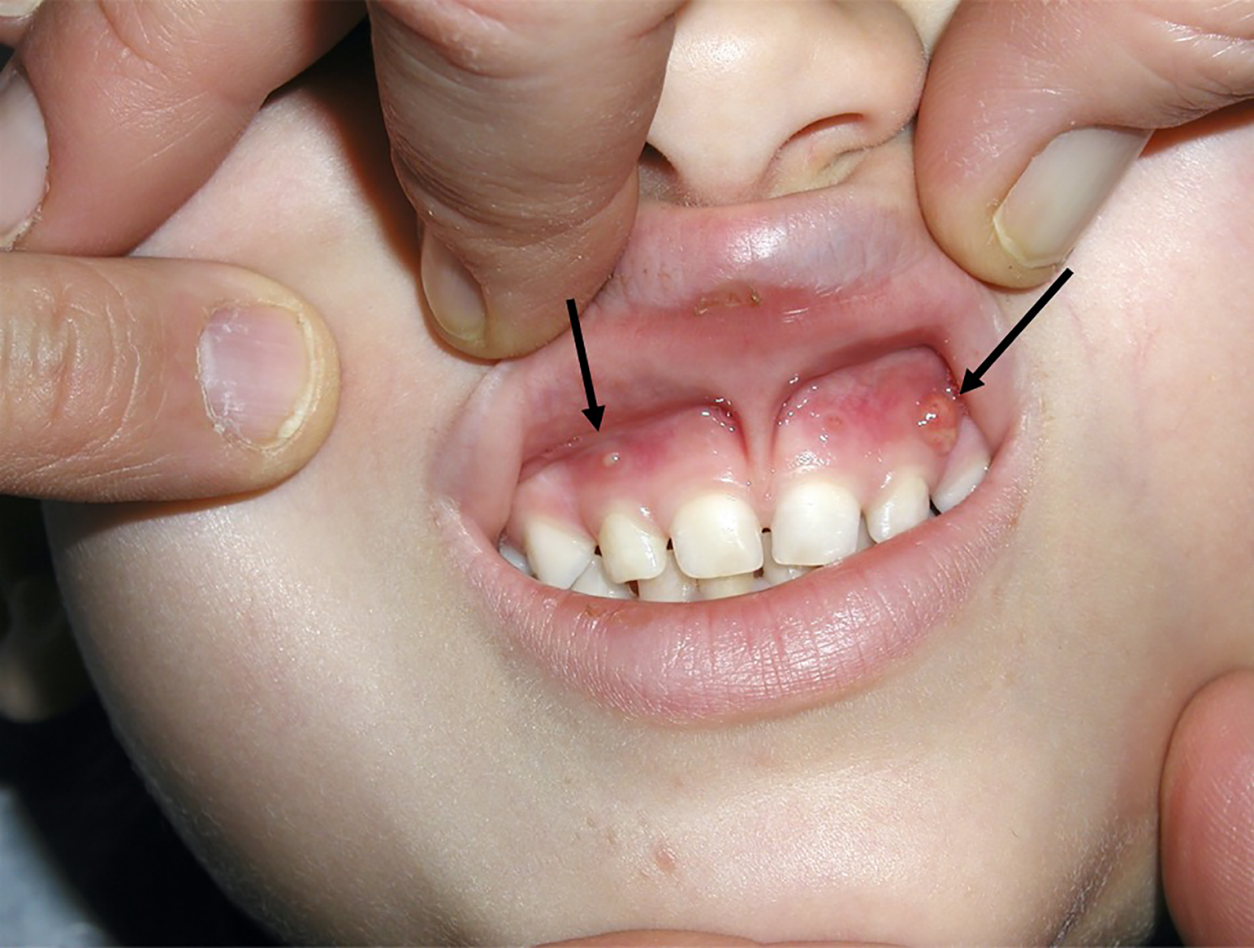
Spontaneous gingival fistulae (black arrow) corresponding to deciduous left maxillary canine and deciduous right maxillary lateral incisor in a 4.3-year-old male patient with XLH.

Typical presentation of patients with XLH includes the hallmarks of rickets and osteomalacia, progressive bowing deformities of the lower limbs, bone pain, stunted growth, and physical dysfunction with reduced daily activities (5, 7, 8). The usual phenotype is shown in **Figure 1**. Approximately two-thirds of patients with XLH show characteristic dental and periodontal lesions, such as spontaneous periapical abscesses with fistulae that develop with no history of trauma or dental decay (9–11). Patients with XLH have enlarged pulps chambers with altered shape and morphology and prominent pulp horns into the tooth crown in primary and secondary molars. These lesions, associated with a poor dentin mineralization, may predispose to recurrent abscesses with fistulae that affect most of the patients. Incisors are affected more than canines and molars (9, 11). A typical periapical abscess with gingival fistula in a patients with XLH is shown in **Figure 2**.

The main skeletal and dental-periodontal abnormalities observed in patients with XLH are summarized in **Table 1**. The clinical manifestations of XLH are variable, ranging from very mild leg deformities to severe systemic involvement. Severity of the phenotype is not correlated to the genotype (3, 12, 13). Delayed motor development and delayed walking may accompany the skeletal signs; they are usually evident in more severe phenotypes. Most of the patients have osteoarticular and muscle pain with weakness, and they frequently show fatigue that reduces exercise tolerance. Fractures are uncommon in pediatric patients with XLH, but osteomalacic fractures (pseudofractures) may affect older patients (8, 14, 15). Moreover, an unexpected increase in mortality in later life has been observed (16).

**Table 1:** Main skeletal and dental-periodontal lesions in patients with XLH.

Cranium	Thorax	Limbs	Total body	Teeth
craniosynostosis scaphocephaly frontal bossing occipital "bullet deformity" delayed anterior fontanel closure° mid facial hypoplasia	costo-chondral junction enlargement (rachitic rosary)	widened wrist, knees, and ankles genu-varum genu-valgum combined genu-varum/valgum short femur tibial torsion^ coxa-vara*	stunted growth disproportionate short stature (short limbs)	abscesses with gingival fistulae^§^ dyschromic enamel

°Rarely; ^in-toeing or ex-toeing; *causing waddling gait; ^§^mainly in incisors and canines, without evidence of trauma or dental decay.

## Biochemical Findings

The hallmark biochemical findings of patients with XLH include hypophosphatemia due to renal wasting of inorganic phosphate and decreased synthesis of 1,25(OH)_2_D. Serum alkaline phosphatase activity is increased in children, but it may be normal in adults. Serum parathyroid hormone (PTH) concentration is normal or slightly elevated (**Table 2**) (4, 5, 7, 8). The association of hypophosphatemia, normal serum levels of calcium and PTH with the evidence of clinical and radiological signs of rickets is suggestive for the diagnosis of XLH.

**Table 2:** Main biochemical findings in patients with XLH.

Parameter	Value
Serum calcium	N
Serum phosphate	↓
Serum alkaline phosphatase	↑
Serum parathyroid hormone	N
Serum 25-hydroxyvitamin D	N
Serum 1,25-dihydroxyvitamin D	↓ or N*
Serum FGF23	N or ↑
Urinary calcium excretion	N
TmP/GFR°	↓

N, normal; ↑, increased; ↓, reduced.

*Inappropriately normal in the setting of hypophosphatemia.

°Maximum tubular reabsorption of phosphate/glomerular filtration rate.

## Radiologic Findings

**Figure 3:**
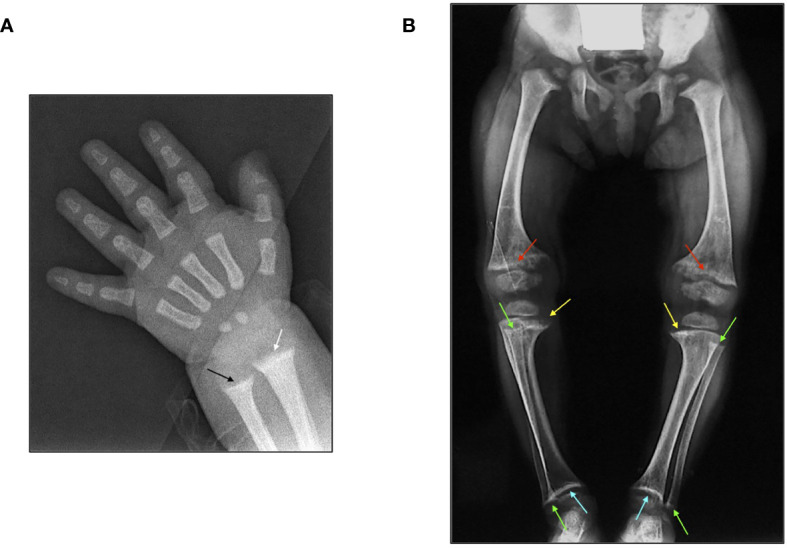
**(A)** X-ray features of the wrist in a 3-month-old male patient affected by XLH at diagnosis: widening and fraying of the epiphyseal plate (white arrow) and metaphyseal concavity of the ulna (black arrow). **(B)** X-ray features of the lower limbs in a 2.5-year-old female patient with XLH at diagnosis: genu-varum and distal medial femoral and tibial bowing with widening and fraying of the distal epiphyseal plate of the femur (red arrow) and the proximal medial epiphyseal plate of the tibia (yellow arrow). Metaphyseal concavity of the proximal and distal fibula (green arrow) and metaphyseal concavity of the distal epiphyseal plate of the tibia (light blue arrow).

X-ray examination is a crucial step for the diagnosis of rickets. Radiographs of the hand and lower limbs show the abnormal growth plates with widened and frayed metaphyses (**Figure 3**). In addition to the diagnosis of rickets, X-ray examination is useful to estimate the severity of rickets by using a score method (Rickets Severity Score, RSS) based on the degree of metaphyseal fraying, concavity, and the proportion of the growth plate affected at the wrist, knee, and ankle (11, 17, 18). The RSS is a 10-point scale, where 10 represents the most extreme degree of rickets severity and 0 represents the absence of radiographic changes of rickets (17, 18). The radiographic response following treatment of nutritional rickets and XLH can be assessed by the RSS, and RSS values correlate with serum alkaline phosphatase activity, a biochemical measure of rachitic activity (17, 18). Recently, the Radiographic Global Impression of Change (RGI-C) score has been validated to estimate the radiographic changes of rickets in children with XLH. RGI-C correlates with clinical, biochemical, and patient-reported changes and represents a complementary radiographic assessment to the RSS. Regional RGI-C scores are assigned for the wrist, knee, and standing long leg based on changes in the specific abnormalities in the later image using a seven-point scale. In the wrist, lucency, fraying and concavity at the metaphyses, and widening of the physes of the distal radius and ulna are rated. In the knee, the same radiographic parameters are assessed at the distal femur and proximal tibia and fibula. The severity of long bone (femur, tibia, fibula) varus and valgus deformity in the bilateral standing long leg images are determined using the same scoring method. The RGI-C scale produces four scores: RGI-C wrist, knee, long leg, and global (19). The medial or lateral parts of metaphysis are more severely affected compared to entire epiphysis in the majority of the patients with XLH. Moreover, skeletal changes are mainly seen in weight bearing joints and therefore upper limb bones are less significantly affected (20).

## Medical Treatment

Conventional treatment of patients with XLH consists of multiple daily doses of inorganic oral phosphate salts associated with vitamin D active analogues, namely calcitriol or alfacalcidol. The short biologic half-life of oral inorganic phosphate salts requires frequent administrations up to six times/day. Compliance with this treatment represents a major issue, mainly in infancy and puberty (8, 15, 21–24).

Conventional treatment, which has been in place for approximately four decades, transiently increases serum phosphate concentration without notable changes in the maximum tubular reabsorption of phosphate normalized to the glomerular filtration rate (TmP/GFR). It is associated with slow improvement in healing of rickets and with residual skeletal deformity in most of the patients. Conventional therapy requires balancing the benefits of treatment with complicated monitoring and potential risks of overtreatment. Gastrointestinal symptoms (diarrhea, bloody stools, and abdominal pain), endocrine abnormalities (hypercalcemia and hyperparathyroidism), and renal complications (hypercalciuria and nephrocalcinosis) may frequently occur in patients receiving long-term conventional treatment (8, 15, 21–24). The effect of conventional treatment in improving linear growth is variable and it has been related to the age at diagnosis and onset of treatment. Nevertheless, the growth response may be unsatisfactory and some patients remain unresponsive (25, 26). Growth retardation in patients with XLH appears to occur in the first few years of life, mainly as a result of the impact of the disease on the growth of the legs (27). Growth response may be associated with different vitamin D receptor promoter haplotypes, providing a possible explanation for some of the clinical variability observed in XLH (28).

Recent studies by using burosumab (Crysvita^®^), a recombinant human IgG1 monoclonal antibody that targets FGF23, showed significant improvements in serum phosphate concentration and TmP/GFR associated with rapid healing of radiologic signs of rickets, reduced osteoarticular and muscular pain, and improved physical function in patients with XLH with RSS >1.5 (29, 30). Furthermore, a randomized, active control, open label, phase 3 trial demonstrated significantly greater clinical improvements in rickets severity, linear growth, and biochemical findings among patients with XLH, with a RSS of at least 2, treated with burosumab compared with patients continuing on conventional therapy (31).

Some data suggested that conventional treatment started in early infancy resulted in improved outcome even though it did not completely normalize skeletal development (25, 32, 33). A recent study showed that patients with XLH had decreased height gain by 1 year of age and remained below population norms thereafter (26). Improved linear growth at week 64 of burosumab treatment in comparison with conventional treatment (31) or prevention of early declines in linear growth in patients aged 1-4 years (30) have been reported in patients with XLH. On the whole, these data seem to indicate that burosumab treatment could be the most appropriate therapy in patients with XLH mainly for a rapid improvement of phosphate metabolism, rickets, and physical function.

Some studies showed that conventional treatment may have a beneficial impact on oral health in patients with XLH (23, 34) depending on the onset, compliance, and duration of the treatment as found in adult patients (8, 35). However, other studies reported that dental and periodontal lesions were incompletely reversed with the conventional treatment (10, 36). Nevertheless, the relation between serum phosphate levels and dental and periodontal abnormalities is not clear. Few data on the effect of burosumab treatment on dental and periodontal lesions are available. A higher frequency of dental abscess in patients with XLH treated with burosumab in comparison with patients receiving conventional treatment might be due to patient variability, or a direct dental benefit of conventional therapy (31).

Longer trials are needed to characterize the long-term effect on linear growth and dental and periodontal lesions in patients treated with burosumab.

Growth hormone (GH) is not a standard treatment in patients affected by XLH with stunted growth. GH treatment may increase short-term linear growth in patients with XLH before puberty (37–43) but it was ineffective in some patients (44, 45). An open uncontrolled study reported that final height improved in a small number of patients with XLH receiving long-term GH treatment in comparison with patients receiving only the conventional treatment (39). A randomized-controlled trial demonstrated that GH treatment resulted in a sustained increase in all linear body dimensions in severely short patients with XLH without a worsening of body disproportion; however, GH treatment did not significantly increase final height compared with controls (45). These data suggest that further studies are needed to closely evaluate the effect of GH treatment on final height in patients with XLH.

## Surgical Treatment

Despite appropriate medical management, patients with XLH are at risk of developing progressive limb malalignments including varus or valgus deformities of the knee. Internal torsion of the tibia and fibula, anteverted femoral neck, patellar dysplasia including chondromalacia and lateral femoral-patellar subluxation may also develop (23). Gait troubles with fatigue are often associated with bone pain and muscular insufficiency at the lower limbs and back (46). Abuse of analgesic drugs may occur in adult patients with XLH to relieve pain (47).

Hemiepiphysiodesis is a minimally invasive technique that may be safely used with good success even in very young children with XLH. The procedure may be repeated in order to maintain the mechanical axis near the center of the knee. Staples represented the surgical procedure of choice for a long time but tension band devices (guided growth) are increasingly being used due to low complication rates and ease of use. By using the latter technique the growth plate is temporarily blocked using a small plate with two screws on the medial or lateral distal femur or proximal tibia. This surgical technique is applicable from the age of 3-4 years and it may be successful until a residual growth is present. Therefore, guided growth must be carried out at least 2–3 years before skeletal maturity (age 14 in girls and age 16 in boys) (48–52). The rationale for the use of guided growth is to correct the deformity at the physis before the occurrence of severe diaphyseal deformity; however, the exact timing for the application of the guided growth techniques may be difficult to identify. Indeed, the exact indications of surgical interventions currently used to correct angular knee deformities remain undetermined and mainly subject to surgeon’s preference (53). Both varus and valgus deformities correct readily and rapidly but genu varum might respond less in adolescents. In growing children guided growth techniques might lead to overcorrection if the plates are left *in situ* for too long. Rebound deformity after plate removal has been reported, although rarely (8). Guided growth should be considered early after 12 months if deformity persists despite maximized medical therapy (8). If the lower limbs deformation is severe the elective procedure is the corrective osteotomy. This is an invasive technique that requires a long hospitalization and a protracted period in which the operated limb cannot be loaded (54, 55).

The use of casts or insoles for the management of lower limb deformity is not recommended in children with XLH (8). Moreover, low extremity bracing is unpredictable and is poorly tolerated mainly in very young patients (48, 56). This procedure has poor effect in correcting and preventing the deformities. Moreover, there is no scientific evidence to support the use of braces to guide growth in patients with XLH (48).

## Multidisciplinary Management

Diagnosis of XLH is generally made by a pediatrician or a pediatric endocrinologist, although the first clinical signs may induce patients to initially seek the advice of an orthopedic surgeon or a nephrologist due to the evidence of impaired phosphate metabolism.

After diagnosis, a coordinating physician is important for the global management of patients with XLH. Usually this task is undertaken by a pediatric endocrinologist or nephrologist. The duty of the coordinator is to monitor the efficacy of the treatment, to prevent the insurgence of side effects, and to seek the advice of other specialists according to the observed complications. Moreover, an important role of the coordinating physician is to offer all advices to the patients and their families.

Strict monitoring of the treatment is the key for clinical success. The efficacy of the treatment is judged upon improvement of linear growth, regression of skeletal malformations, including genu-varum or genu-valgum, and improvement in quality of life (QoL). Biochemical findings, as reported in **Table 2**, should be periodically assessed in order to prevent the complications associated with over or under-conventional treatment dosages.

The main complications of patients with XLH during childhood and adolescence are summarized in **Table 3**. Some of these are related to the disease itself or to the occurrence of side effects related to the conventional treatment.

**Table 3:** Main complications reported in patients with XLH according to specialized subdisciplines (in alphabetic order).

Subdisciplines	Complications
Endocrinology	related to conventional treatment: - secondary hyperparathyroidism - tertiary hyperparathyroidism - obesity (?) stunted growth
Nephrology	related to conventional treatment: - hypercalciuria - nephrocalcinosis - nephrolithiasis
Neurosurgery	symptomatic craniosynostosis symptomatic Arnold-Chiari malformation medullary cervical and thoracic syringomyelia
Odontostomatology	recurrent spontaneous abscesses with gingival fistulae in both deciduous and permanent teeth maxillofacial cellulitis
Ophthalmology	related to craniosynostosis: - proptosis - elevation of the optical nerve head - papilledema
Orthopedics	genu-varum/valgum or combined lower limbs deformities including severe asymmetry
Otolaryngology	hearing loss (mostly neurosensorial)
Psychology	difficulties in social and affective relationships fear of undergoing surgery deterioration in quality of life
Rheumatology	osteoarticular pain muscular pain

### Endocrinological Complications

Possible endocrine complications are the development of secondary or tertiary hyperparathyroidism. Secondary hyperparathyroidism is due to an insufficient replacement with active vitamin D metabolites or to an excessive dose of inorganic phosphate salts. It is usually reversed by optimizing treatment (23, 57, 58). Tertiary hyperparathyroidism is a severe complication of the conventional treatment and it is generally irreversible; it may require total parathyroidectomy to control hypercalcemia (59, 60).

Overweight or obesity affect approximately one-third of patients with XLH (61). Obesity could be associated with impairment of glucose and lipid metabolism but pathogenetic factors are undefined (8, 61). Adolescent and adult patients with XLH are prone to develop obesity partly because rheumatological and chronic bone complications decrease the propensity of patients to exercise (8). A long duration of conventional treatment has been reported to be a main determinant in developing progressive overweight (8, 24, 61). However, further studies are needed to clarify the progressive weight gain during treatment.

Despite the treatment, stunted and disproportionate growth is evident in early childhood and persists through adolescence and adulthood in many patients. This may cause psychological distress both in the patient and in the parents, heavily impairing QoL.

### Renal Complications

Conventional treatment with oral phosphate and active vitamin D metabolites may expose patients with XLH to the occurrence of nephrocalcinosis and later on of nephrolithiasis. Signs of nephrocalcinosis have been reported in 30%-70% of pediatric patients (5, 8, 22, 23, 62). There is evidence of a direct relationship between the daily dose of inorganic phosphate salts and the risk of renal complications (5, 8, 62). Periodic assessment of the renal parenchyma is therefore mandatory. Recent guidelines recommend ultrasonographic evaluation every 1-2 years in patients without signs of nephrocalcinosis, and yearly in patients with renal disease or persistent hypercalciuria. Nephrocalcinosis requires the nephrologic assessment (8, 24, 56).

### Neurosurgical Complications

Craniosynostosis due to altered growth and fusion of the osteomalacic head bones may occur early in patients with XLH. XLH has been associated with synostosis of the coronal, lambdoid and sagittal sutures, including pansynostosis, but it is believed to most commonly affect the sagittal suture (63). The clinical signs associated with craniosynostosis are increased intracranial pressure (with headache reported by older children), vomiting, papilledema, strabismus, or pulsating anterior fontanelle. Moreover, proptosis and elevation of the optical nerve head may be ocular complications of craniosynostosis (64–66).

**Figure 4:**
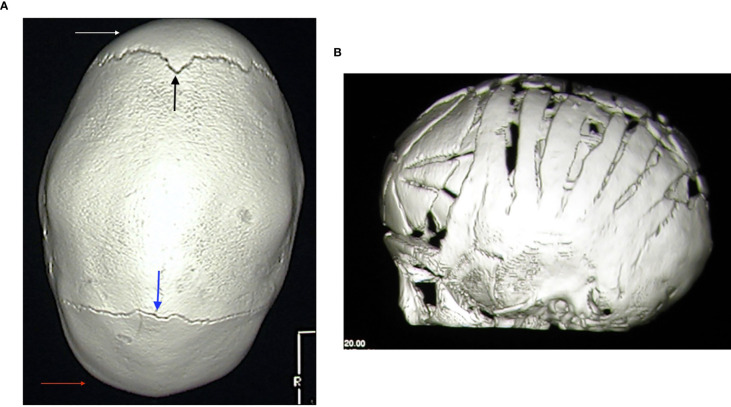
**(A)** 3D-reformatted axial computed tomography scan showing fusion of the sagittal suture and classic scaphocephalic head shape in a 3.2-year-old male patient with XLH. Frontal (white arrow) and occipital (red arrow) bossing with open coronal (black arrow) and lambdoid (blue arrow) sutures is also evident. **(B)** 3D-reformatted axial computed tomography scan showing the neurosurgical correction of scaphocephaly in a 8-month-old male patient with XLH.

Craniosynostosis can be observed in about one-third of patients with hypophosphatemia but only a minority of patients require surgery (67). **Figure 4** shows the typical scaphocephalic head shape in a patient with XLH and the surgical approach in order to correct cranyosinostosis. Therefore, all patients with XLH need a strict monitoring of cranial morphology and circumference, conformation of the anterior fontanelle, as well as the neurologic development, mainly during the first year of life.

**Figure 5:**
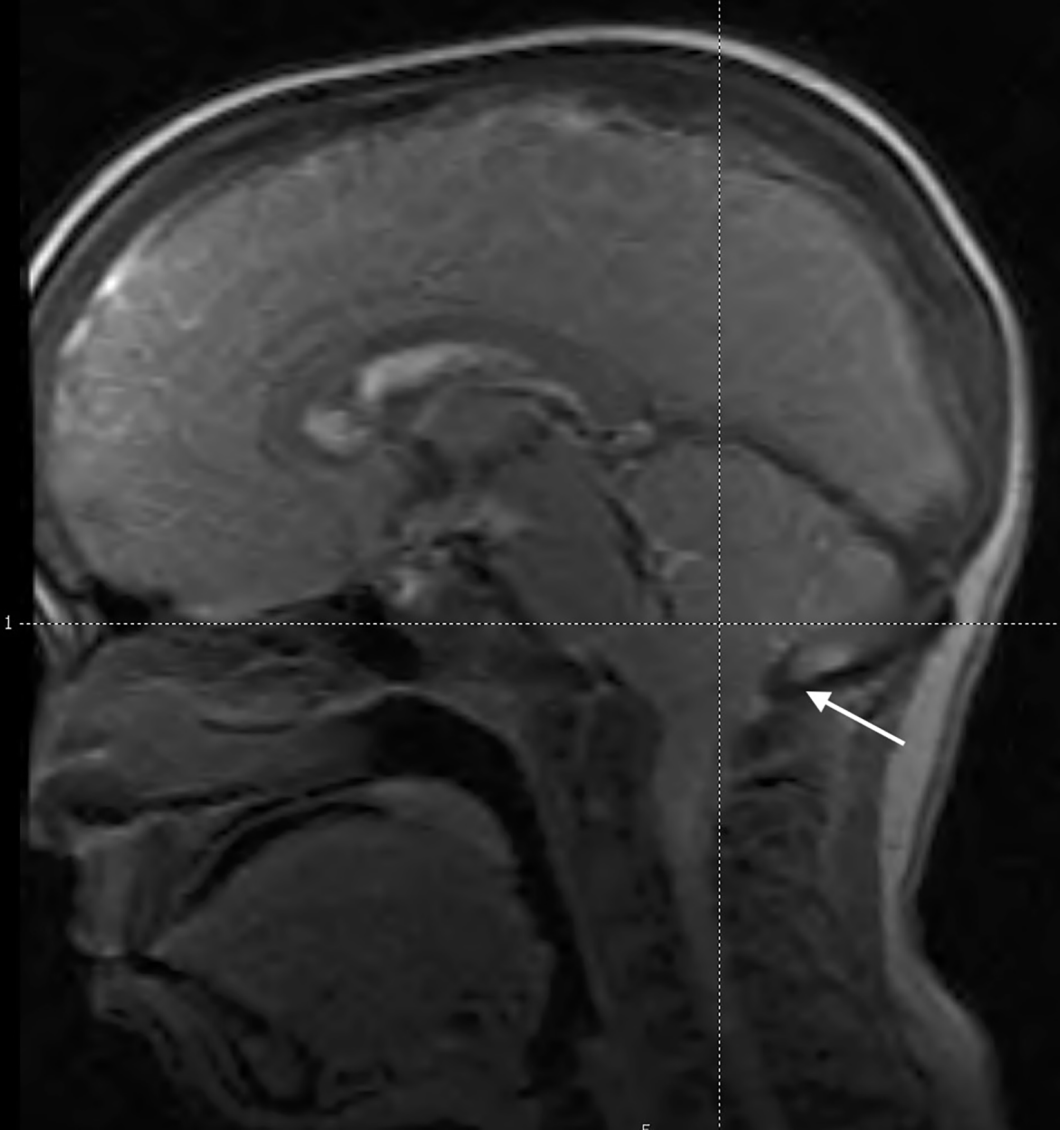
Midsagittal magnetic resonance image of the head in an 11.5-year-old male patient with XLH showing caudal descent of the cerebellar tonsils through the foramen magnum (Arnold-Chiari 1 malformation) (white arrow). He reported transient headache. Scaphocephaly was also evident.

Rothenbulhler et al. (68) reported a complete or partial fusion of the sagittal suture in 59% of patients with a protrusion of the cerebellar tonsils in 25%. The development of Arnold-Chiari malformation may be associated with severe headache and vertigo, which may require neurosurgery if symptomatic (23). Nevertheless, only a small proportion of patients with XLH (<10%) requires neurosurgery (68). Furthermore, cervical and thoracic syringomyelia may develop in some patients (8, 68–70). The typical picture of Arnold-Chiari malformation in a patient with XLH is reported in **Figure 5**.

### Odontostomatologic Complications

Approximately two-thirds of patients with XLH have dental and periodontal lesions of both deciduous and permanent teeth (8, 11, 23). Spontaneous abscesses are caused by an abnormal dentin mineralization that results in a widened predentin zone and, consequently, as the characteristic interglobular dentin, reflecting an inability of mineralization foci (calcospherites) to fuse into a unified mineralization front (9, 34, 71–73). Although the *PHEX* genotype may affect dentin mineralization and morphology of pulp chambers, *PHEX* gene mutations did not correlate with dental phenotype and disease severity (11). Enamel discoloration and an aberrant cementum phenotype have been found in both Hyp mice and patients with XLH (74, 75). Thus, an impairment of all the three hard tissues of the tooth (enamel, dentin, and cementum) characterizes the dental phenotype in patients with XLH, being dentin structure and mineralization the most severely damaged.

Dental examination should be performed at least twice yearly in patients with XLH (8). Pit and fissure sealant and maintenance of good oral hygiene are highly recommended (36). Acute abscesses may require antibiotic treatment depending on the extent and severity of the infection. The decision to extract or treat endodontically the deciduous teeth will depend on the extent of the infection, recurrence and the expected timing of normal exfoliation of the permanent tooth. Endodontic treatment or re-treatment of the permanent teeth are the preferred options, although healing after endodontic treatment might not be as favorable as in healthy patients (8).

### Orthopedic Approach

Regular orthopedic follow-up is recommended in patients with XLH. Early medical treatment is essential for a successful outcome. However, in many patients it is not able to arrest the progression of the skeletal deformities of the lower limbs. Legs deformities are usually associated with waddling gait, reduced motor abilities, osteoarticular pain, and articular degeneration later in life (5, 7, 8, 23).

Persisting skeletal deformities despite optimized medical treatment and/or the presence of symptoms interfering with function should be considered for surgical treatment (8). Elective surgical treatment by a surgeon with expertise in metabolic bone diseases should be performed only in patients in whom conventional treatment has been maximized for at least 12 months (8).

### Physiotherapeutic Approach

Physiotherapy is recommended in patients with XLH mainly after orthopedic surgery (8, 23, 56). Skeletal symptoms are a contributing factor for the reduced motor ability in these patients. Personalized activities to reduce osteoarticular and muscular pain and to facilitate movements are suggested in order to improve QoL. Osteoarticular pain is highly prevalent among adults and children. Nearly all adults (97%) and the majority of children (65%-80%) with XLH (5, 76) experience bone and/or joint pain. Muscle pain has also been reported in 63% of adult patients and 60% of children with XLH (76). Many adolescent and adult patients with XLH refer a self-therapy by using various analgesic medications to reduce pain at lower limbs (5, 76). Some patients require rheumatologic examination for the prescription of a personalized antalgic treatment. Analgesics were taken at least once a week by 67% of adult patients with XLH (76).

### Otolaryngoiatric Complications

Adult patients with XLH may develop a hearing loss of varying degrees, but its prevalence is uncertain (14). Some patients suffer tinnitus and vertigo associated with low frequencies hearing loss, as found in patients with Meniere’s disease (77). Sensorineural hearing loss, often asymmetric, appears most prominent, but a mixed picture thought to be caused by endolymphatic hydrops has also been found (78). Some patients may have generalized osteosclerosis and thickening of the petrous bone (14, 79, 80). In the *Phex* mouse model the treatment did not have any impact on endolymphatic hydrops, hearing or vestibular dysfunction (81). Hearing loss is rarely seen in pediatric patients (77, 79). A consensus statement recommended hearing test from the age of 8 years in pediatric patients if hearing difficulties are suspected (8). Treatment is similar as for other causes of hearing loss including hearing aids, prevention of noise exposure, and avoidance of ototoxic drugs (8).

### Quality of Life

Few data are available on QoL and social impact for patients with XLH during childhood (5). A recent phase 3 trial study in children aged 5-12 years with XLH showed that switching from conventional treatment to burosumab therapy improved some measure of patient-reported outcomes as well as physical health score (82). Studies in adults with XLH showed a progressive reduction of QoL with the progression of the disease (22, 47, 83). Some adult patients described the symptoms as having impact on their mood/mental health, relationships, social life and leisure activities (83). Symptoms had often worsened over time, and for many, they were associated with concern about the future. Most patients were worried or felt guilty about having children with XLH (47). The occurrence of enthesopathies is associated with a worse QoL (83). A recent review showed that the level of healthcare resource utilization among adults with XLH was indicative of substantial socio-economic burden and that they may not receive appropriate care and treatment; a possible explanation for this is a lack of awareness among healthcare professionals (84).

## Conclusions

XLH is a rare genetic, multisystemic, and invalidating disorder affecting children and adults that requires a multidisciplinary approach. XLH severely impacts QoL. There is evidence that patients with XLH should be supported by a team of experts in order to improve the criteria for diagnosis and treatment regimen. Although long-term effects of burosumab treatment on final height, lower-limb deformities, craniosynostosis, dental abnormalities, and disability are not yet known, phosphate metabolism, rickets, bone health, and QoL are markedly improved in both pediatric and adult patients with XLH by treatment with burosumab. Nevertheless, further studies are needed to compare the efficacy of different treatments, particularly during infancy and adolescence.

## Author Contributions

Both the authors have contributed equally to design, write, and discuss all the sections of the manuscript, and share first authorship. All authors contributed to the article and approved the submitted version.

## Conflict of Interest

The authors declare that the research was conducted in the absence of any commercial or financial relationships that could be construed as a potential conflict of interest.
